# The 21st century marks a rise in TURP retreatment rates: an analysis of Veterans Health Administration data

**DOI:** 10.1007/s00345-025-06083-5

**Published:** 2025-11-17

**Authors:** Sirpi Nackeeran, Kylie M. Morgan, Henry Chen, Jennifer T. Anger, Roger L. Sur, Tyler Sheetz

**Affiliations:** 1 Department of Urology, University of California San Diego, San Diego, CA USA; 2 Department of Radiation Medicine and Applied Sciences, University of California San Diego, La Jolla, San Diego, CA USA; 3 VHA San Diego Health Care System, La Jolla, San Diego, CA USA; 4 Still University School of Osteopathic Medicine, Mesa , AZ USA

**Keywords:** Benign prostatic hyperplasia, Transurethral resection of prostate, Surgical trends

## Abstract

**Purpose:**

With the advent of new and emerging procedures for benign prostatic hyperplasia (BPH), as well as a greater reliance on medical therapies, trainees may be receiving less exposure to the longstanding gold standard of treatment, transurethral resection of the prostate (TURP). Although TURP outcomes are generally durable, success rates vary. We sought to perform a descriptive analysis of temporal trends in TURP outcomes, evaluating whether those performed prior to 2001 were less likely to require re-operation compared to more contemporary TURP procedures.

**Methods:**

We accessed the Veteran’s Affairs (VA) national repository of electronic medical record data. We included veteran males with no prior BPH surgery who received a TURP. We used billing codes to determine retreatment and complications rates within 5 years of TURP. Outcomes were assessed via logistic regression analysis to compare TURP outcomes from before and after the year 2001. Comparisons between variables were done via Chi-Square test.

**Results:**

2,084 underwent TURP prior to 2001 and 35,817 underwent TURP after 2001. Patients undergoing TURP after 2001 were more likely to undergo new procedures within 5 years (15.4% vs. 10.3%, OR 1.49, 95% CI 1.29–1.73, *p* < 0.001) and be on BPH medications postoperatively (65% vs. 30%, *p* < 0.001). However, these post-2001 patients were also less likely to have incontinence (15.5% vs. 19.7%, OR 0.60, 95% CI 0.53–0.67, *p* < 0.001).

**Conclusions:**

TURP prior to 2001 was associated with lower reoperation rate or continue medical therapy, however it was associated with higher rates of incontinence. Several potential explanations exist for these trends, and future studies are necessary to elucidate the reasons for them.

**Supplementary Information:**

The online version contains supplementary material available at 10.1007/s00345-025-06083-5.

## Introduction

Benign prostatic hyperplasia (BPH) is a prevalent condition common among middle-aged and older men, with its incidence increasing over time [1]. BPH arises from the proliferation of stromal and epithelial cells in the transition zone of the prostate, resulting in urethral narrowing and increased prostatic volume and stromal muscle tone [2, 3]. The non-malignant enlargement of the prostate gland often compresses the urethra, leading to lower urinary tract symptoms (LUTS), which include obstructive and irritative voiding issues that significantly impact quality of life [2, 4].

Management options for BPH vary and include observation [5], alpha-blockers, 5-alpha reductase inhibitors, and phosphodiesterase type 5 inhibitors [6]. Since the invention of the resectoscope in 1926, transurethral resection of the prostate (TURP) has been the gold standard for surgical management of BPH [7]. Several new technologies have since been developed, with varying outcomes and side-effect profiles. Due to increased usage of medical management as well as the abundance of other technology available, the use of TURP has decreased over time [8].

Despite TURP’s widespread acceptance as the gold standard treatment for BPH, there is limited knowledge or validation on the optimal amount of prostatic tissue that should be removed to achieve the best functional outcome. It has been previously posited that changes in surgical technique have made outcomes less durable over time with surgeons resecting less aggressively [8]. This trend may coincide with the advent of BPH guidelines establishing medical management as first line therapy in 1994[9] and the introduction of the bipolar TURP in 2001.

Our study investigates whether the era within which a TURP was performed was associated with reoperation rates or surgical side effects. To do so, we turned to the Veteran Affairs Informatics and Computing Infrastructure (VINCI), which collects electronic health record data from Veterans Affairs (VA) across the United States. Due to a greater proportion of patients failing medical management prior to surgery and the availability of several new surgical modalities, we hypothesized that TURPs performed prior to the year 2001 might be associated with lower reoperation rates. By identifying potential associations between year of surgery and re-treatment rates or complications, we hope our findings will shed light on BPH practice patterns and better inform physicians counseling patients on the likely outcomes when offering TURPs.

## Methods

### Data source

This study was conducted in accordance with the Department of Veterans Affairs requirements and received Institutional Review Board approval. We accessed data through the Veterans Affairs Informatics and Computing Infrastructure (VINCI), the VA’s national repository of data collected from its electronic medical record system. VINCI contains information logged for all patient encounters, including demographics, labs, diagnosis and procedure billing codes, and medications based on pharmacy prescriptions filled. International Classification of Diseases (ICD) revision 9-Clinical Modification (CM) and revision 10-CM were used to diagnosis BPH. Current Procedure Terminology (CPT), Healthcare Common Procedure Coding System (HCPCS), and pharmaceuticals were utilized for this study.

### Cohort selection

We collected data on adult men with no prior BPH surgery in the VA system who received a TURP. CPT and ICD codes with their corresponding procedures are listed in Supplementary Table 1. We considered any patient who had previously had a CPT (52601, 52612), ICD-9 (60.2, 60.29), or ICD-10 (0VT08ZZ) procedure code to have had a TURP. CPT codes used to identify other prior BPH surgeries were as follows: Greenlight photovaporization of the prostate (PVP, 52648), transurethral incision of the prostate (TUIP, 52450), Urolift (52441), Rezum Water Vapor therapy (53854), holmium laser enucleation of the prostate (HoLEP, 52649), and simple prostatectomy (55821, 55831). All patients with a BPH procedure recorded prior to their first TURP were excluded. ICD-9 and ICD-10 diagnosis codes were used to identify the following patient characteristics pre-operatively: urinary retention (R33.9, 788.20, 788.21, 788.29), urethral stricture (N35.01, N35.11, N35.81, N35.91), urinary incontinence (N39.3, N39.4, N39.41, N39.42, 788.32, 788.31, 788.34). Patients were considered to be on medical therapy for BPH if they were recorded to have used tamsulosin, alfuzosin, doxazosin, finasteride, or dutasteride based on filled pharmacy prescriptions. Charlson comorbidity index was calculated for each patient, and race, ethnicity, marriage status, and body mass index (BMI) were imported from demographic and vital signs information recorded for each patient. Race and ethnicity were combined for purposes of analysis.

### Outcomes

Our primary outcome of interest was occurrence of a BPH procedure within 5 years of first time TURP. Secondary outcomes included occurrence of all urinary incontinence or specifically stress urinary incontinence. Additional variables assessed as postoperative outcomes at 5 years included: urge incontinence, mixed incontinence, insensate incontinence, urethral stricture, refill of BPH medications, or refill of catheter supplies according to HCPCS code (A4310-16, A4351-4, A4357-8, A4338, A4340, A4344, A4346, A5102, A5112). 30-day and 90-day outcomes were assessed for diagnosis of urinary tract infection (N39.0, 599.0), blood transfusion (CPT 36430), or clot evacuation procedure (CPT 52001). Urinary retention diagnosis codes were not assessed due to persistent use of this code after successful TURPs.

### Statistical analysis

We used a chi-square test to compare categorical variables in Tables 1 and 2, and non-parametric tests to compare continuous variables. We used multivariable logistic regression where alpha = 0.05 to assess outcomes of interest comparing patients who received a TURP between 2001 and 2024 against those who received a TURP between 1996 and 2000. We adjusted for age at time of TURP, pre-operative urinary retention diagnosis, pre-operative urethral stricture, pre-operative urinary incontinence, race/ethnicity, Charlson comorbidity index (CCI), and BMI.

**Table 1:** Baseline characteristics of VA patients 1996–2000 and 2001–2024

	1996–2000	2001–2024	*p*-value
*N*	2084	35,907	
Age (median years, [IQR])	73 (66,78)	71 (65,78)	*0.038*
Pre-op urinary retention (n, %)	756 (36.3%)	14,764 (41.1%)	*< 0.001*
Pre-op urethral stricture (n, %)	8 (0.4%)	64 (0.2%)	*< 0.001*
Pre-op urinary incontinence (n,%)	116 (5.6%)	3829 (10.7%)	*< 0.001*
Pre-op medication (n,%)	520 (25.0%)	266,946 (75.0%)	*< 0.001*
Marital status (n, %) Divorced Married Never married Separated Single Unknown Widow	422 (20.2%) 995 (47.7%) 167 (8.0%) 50 (2.4%) 3 (0.1%) 52 (2.5%) 395 (19.0%)	9001 (25.1%) 18,326 (51.0%) 2971 (8.3%) 1009 (2.8%) 18 (0.1%) 301 (0.8%) 4281 (11.9%)	*< 0.001*
Race and ethnicity (n,%) Black Hispanic/Latino Not Hispanic White Other Unknown	238 (11.4%) 79 (3.8%) 974 (46.7%) 77 (3.7%) 716 (34.4%)	5783 (16.1%) 2032 (5.7%) 24,624 (68.6%) 833 (2.3%) 2635 (7.3%)	*< 0.001*
Charlson Comorbidity Index (n, %) 0 1 2+ Unknown	934 (44.8%) 481 (23.1%) 190 (9.1%) 479 (23.0%)	11,429 (31.8%) 10,690 (29.8%) 8317 (23.2%) 5471 (15.2%)	*< 0.001*
Body Mass Index (n, %) *≤*30 >30	1711 (82.1%) 373 (17.9%)	27,523 (75.9%) 8654 (24.1%)	*< 0.001*

## Results

We collected data on 37, 991 adult male Veterans between 1996 and 2024. Baseline characteristics are represented in Table 1. Pre-2001 TURP patients were likely to be healthier and less obese, with a higher proportion of patients with a Charlson comorbidity index of 0 (44.8% vs. 31.8%, *p* < 0.001), and a lower proportion with BMI > 30 (17.9% vs. 24.1%). Only 25% of pre-2001 patients were on BPH medications prior to their procedure, as opposed to 75% in the 2001–2024 group (*p* < 0.001). Patients treated before 2001 were also less likely to be diagnosed preoperatively with incontinence (5.6% vs. 10.7%, *p* < 0.001) or urinary retention (36.3% vs. 41.1%, *p* < 0.001).

Frequencies of post-operative outcomes at 5 years are represented in Table 2. Post-operatively, patients treated before 2001 were less likely to have a new BPH procedure (10.3% vs. 15.4%, *p* < 0.001) or start medications (30.0% vs. 65.0%, < 0.001)within 5 years of their TURP While pre-2001 patients had higher rates of urinary incontinence of all types (19.7% vs. 15.5%, *p* < 0.001), prevalence of postoperative stress urinary incontinence diagnosis was similar (2.2% vs. 2.8%, *p* = 0.095). There were no differences in urethral stricture rates or refills of catheter supplies.

**Table 2:** Post-operative outcomes

	1996–2000	2001–20,024	*p*-value
*N*	2084	35,907	
New procedure within 5 years (n, %)	214 (10.3%)	5516 (15.4%)	*< 0.001*
All incontinence (n, %)	410 (19.7%)	5566 (15.5%)	*< 0.001*
Stress incontinence (n, %)	45 (2.2%)	1006 (2.8%)	0.095
Urge incontinence (n, %)	112 (5.4%)	2344 (6.5%)	*0.042*
Mixed incontinence (n, %)	27 (1.3%)	774 (2.2%)	*0.010*
Insensate incontinence (n, %)	8 (0.4%)	413 (1.2%)	*0.002*
Other incontinence (n, %)	339 (16.3%)	4460 (12.4%)	*< 0.001*
Urethral stricture (n, %)	5 (0.2%)	69 (0.2%)	0.822
Refill of catheter supplies (n, %)	86 (4.1%)	1651 (4.6%)	0.343
Medications within 5 years (n, %)	625 (30.0%)	23,331 (65.0%)	*< 0.001*

Table 3 represents the multivariable logistic regression analysis comparing the 2001–2024 group against the 1996–2000 group and adjusting for age, pre-operative urinary retention, urethral stricture, or incontinence, race/ethnicity, CCI, and BMI > 30. The results showed that patients who had their first TURP after 2000 were more likely to require another BPH procedure within 5 years (OR 1.49, 95% CI 1.29–1.73, *p* < 0.001). Older age, pre-operative urinary retention, and CCI *≥* 2 were other factors identified within the model to be associated with a repeat BPH procedure.

**Table 3:** Multivariable logistic regression assessing associations between TURP year and outcomes adjusting for preoperative patient characteristics

Outcome	Characteristic	OR (95% CI)	*p*-value
BPH procedure within 5 years	TURP year 2001–2024^*^	1.49 (1.29, 1.73)	*< 0.001*
	Age (years)^+^	1.01 (1.00, 1.01)	*< 0.001*
	Urinary retention^^^	1.20 (1.14, 1.28)	*< 0.001*
	Urethral stricture	1.40 (0.76, 2.42)	0.200
	Incontinence	1.08 (0.98, 1.18)	0.110
	Race Non-Hispanic White Black/African American Hispanic/Latino Other Unknown	*Ref* 1.08 (1.00, 1.17) 1.10 (0.97, 1.24) 0.85 (0.69, 1.02) 0.79 (0.71, 0.89)	0.052 0.130 0.093 *< 0.001*
	Charlson comorbidity index 0 1 2+ Unknown	*Ref* 0.98 (0.92, 1.06) 0.93 (0.86, 1.01) 1.02 (0.94, 1.12)	0.700 0.0669 0.600
	BMI > 30^#^	0.94 (0.88, 1.01)	0.072
All urinary incontinence at 5 years	TURP year 2001–2024^*^	0.60 (0.53, 0.67)	*< 0.001*
	Age (years)^+^	0.99 (0.99, 1.00)	*< 0.001*
	Urinary retention^^^	1.61 (1.52, 1.71)	*< 0.001*
	Urethral stricture	1.16 (0.65, 2.00)	0.600
	Incontinence	5.20 (4.84, 5.60)	*< 0.001*
	Race Non-Hispanic White Black/African American Hispanic/Latino Other Unknown	*Ref* 0.96 (0.88, 1.04) 0.98 (0.86, 1.11) 1.12 (0.93, 1.34) 0.76 (0.68, 0.85)	0.300 0.700 0.200 *< 0.001*
	Charlson comorbidity index 0 1 2+ Unknown	*Ref* 0.98 (0.91, 1.05) 0.83 (0.76, 0.89) 0.88 (0.80, 0.97)	0.600 *< 0.001* *0.007*
	BMI > 30^#^	1.17 (1.10, 1.26)	*< 0.001*

^* Denotes comparison against patients who received TURP between 1996–2000^

^+ Denotes continuous variable^

^^ Denotes comparison against lack of diagnosis or characteristic^

^# Denotes comparison against BMI *≥*30^

In our multivariable regression analysis assessing secondary outcomes, 2001–2024 TURP patients were less likely to have urinary incontinence of all types (OR 0.60, 95% CI 0.53–0.67, *p* < 0.001). Results when selecting for the stress incontinence diagnosis code only were not statistically significant.

## Discussion

The emergence of new technologies for bladder outlet obstruction surgery has led to a diminishing role for the TURP, which has long been the gold standard of the field. As the utilization of TURP surgeries has changed over time, we had hypothesized that more recent TURPs may be associated with higher rates of reoperation. Through an examination of the VA’s national database, we aimed to provide perspective for urologists seeking to define the role of TURP going forward, including its strengths and weaknesses in the modern era. We found that TURPs performed after the year 2000 did, in fact, have a higher risk of requiring a repeat procedure within 5 years, however they were also associated with a significantly lower incontinence (all types) risk. The results of this study will allow for updated counseling regarding the durability of TURPs and their risk profile and will also better inform urologists seeking to define the role of the TURP within the context of bladder outlet obstruction surgeries they offer S1.

There is evidence that TURP utilization continues to decrease, as seen in a recent retrospective study which showed a decline in the percentage of bladder outlet obstruction procedures that consisted of TURP, from 92.1% to 71.9% between 2002 and 2014[10]. It has been previously posited that TURPs are being used less frequently due to the rapid increase in use of medical therapies for BPH [11]. Izard et al. suggest that the patients being selected for TURP have higher risk of failure or complications due to their inherent pre-existing failure of medical therapy. This finding is in line with the results of the present study, given the significantly higher usage of medical therapies in the post-2000 group noted in our results. However, this increased usage may also be at least partially attributable to increasing usage of BPH medications in general at the turn of the century. In a single center retrospective study in China by Peng et al., similar patterns were observed [12]. Over an 11-year period, patients with BPH were more likely to opt for medical therapy and less likely to elect for surgery, however those that underwent surgery did not have significant differences in complication rates.

Furthermore, the advent of a wide array of bladder outlet obstruction surgical technologies has likely decreased utilization of TURP, with each technique offering varying strengths and weaknesses with tailored AUA guidelines [13]. Patients seeking to preserve ejaculation now have the options of Rezum, Urolift, and Aquablation [14–16]. For patients with higher bleeding risk, greenlight photovaporization of the prostate has been proposed as a potentially safer option [17]. Patients with large prostates have the option of HoLEP or simple prostatectomies, while patients seeking less invasive options are able to seek out prostatic artery embolization [18, 19]. The plethora of options could have also contributed to the earlier adoption of a second BPH surgery in recent years that was noted within the present study, as patients may have been more willing to try an alternate surgical modality rather than repeating a TURP.

Other studies have also sought to answer the degree to which surgeon experience and training influence the ability to perform a TURP. A prospective study by Cury et al. revealed that a senior urologist was able to resect prostate tissue at four times the rate of urologists who had only performed 25 and 50 TURPs [20]. Although time of resection could not be measured within this study, it stands to reason that as urologist are performing less TURPs over time, their experience and therefore speed of resection may lead to resection of less prostatic tissue.

This study was not without limitations. As with all retrospective studies reliant on claims data, we could not eliminate the possibility that diagnosis codes were being applied inaccurately or inappropriately to patients. Specifically, we could not verify whether certain pre-operative diagnoses, such as urinary retention, were also applied at post op visits despite the resolution of the original diagnosis. Furthermore, we lacked granularity in the data that could have provided additional context to the diagnoses and procedures involved. Pre-operative voiding studies, prostate size, and medications would have helped adjust for differences in patient selection across time. Intraoperative details such as operative time or grams resected would have additionally allowed us to quantify changes in surgical technique. Additionally, we could not account for changes in practice patterns over time influencing results. In 2015, which was within the time span of this study, the United States also transitioned to the ICD-10 coding system. Medication data may also have been limited prior to the year 2000 as certain medication codes were not available for analysis prior to this year due to their lack of Food and Drug Administration approval. Finally, the infrastructure of the VA electronic health record matured substantially after 2000, leading to more complete documentation of diagnoses. Our study could not characterize whether each TURP was completed with monopolar or bipolar energy. Interestingly, results were not statistically significant when selecting only the stress urinary incontinence code as an outcome. Overall, incontinence diagnosis codes can be unreliable, which limits the interpretation of our incontinence results. It is important to note that the post-2001 population was younger, more likely to present in urinary retention, less obese, and have more comorbidities. All of these factors reflect different referral patterns and were potential confounders that explain the higher retreatment rates and lower incontinence rates. On the other hand, this study greatly benefited from a large sample size drawing from VA institutions across the United States. Demographic information was utilized and we were able to consider social factors. Robust statistical methods were used to control for several possible confounding factors and the associations of primary and secondary outcomes were strong.

In summary, we showed that TURPs performed after the year 2000 were associated with an additional bladder outlet obstruction procedure within 5 years and also with a lower rate of urinary incontinence. This study suggests that TURPs may have become less fraught with adverse events at the expense of durability. While suspected, this finding had never been established in the literature. Urologists can look to these results to better inform the way they counsel patients regarding the success rates of TURPs. Furthermore, physicians offering a multitude of BPH procedures will be able to better define the specific role that TURPs can play for their patients. Future studies can expand upon these findings by prospectively examining specific factors of each TURP in a standardized fashion, such as degree of resection to capsule, or patency of the lumen of the prostatic urethra.

## Conclusion

Compared to patients in the year 2000 or earlier, patients undergoing modern TURP are more likely to experience a repeat BPH procedure within 5 years, but less likely to have urinary incontinence. These findings merit further investigation as to why more recent TURP procedures pose a higher risk of re-intervention. All stakeholders of healthcare would benefit from understanding the root cause of these findings.

Supplementary Table 1. Diagnosis, medication, supply, and procedure codes with corresponding definitions.

## Supplementary Information

Below is the link to the electronic supplementary material.

Supplementary material 1 (DOCX 14.0 kb)

## Data Availability

The data analyzed in this study were obtained from the U.S. Department of Veterans Affairs national electronic medical records repository. Due to federal regulations and institutional policies, the data are not publicly available but may be accessible upon reasonable request and with appropriate VA approvals.
