# Supplementary material for: The 21st century marks a rise in TURP retreatment rates: an analysis of Veterans Health Administration data
Source: World J Urol. 2025 Nov 17;43(1):701. doi: 10.1007/s00345-025-06083-5 (PMC12628495; doi:10.1007/s00345-025-06083-5)
Supplement: Supplementary file 1 — Supplementary material 1 (DOCX 14.0 kb) [file 345_2025_6083_MOESM1_ESM.docx]

Supplementary Table 1. Diagnosis, medication, supply, and procedure codes with corresponding definitions.

| Tamsulosin | G9503 |  |
| --- | --- | --- |
| Finasteride | S0138 |  |
| TURP procedure | 52601 | Transurethral electrosurgical resection of prostate, including control of postoperative bleeding, complete (vasectomy, meatotomy, cystourethroscopy, urethral calibration and/or dilation, and internal urethrotomy are included) |
|  | 52612 | Transurethral resection of prostate; first stage of two-stage resection (partial resection) |
|  | 52614 | Transurethral resection of prostate; second stage of two-stage resection (resection completed) |
|  | 52620 | Transurethral resection; of residual obstructive tissue after 90 days postoperative |
|  | 52630 | 52630 Transurethral resection; of regrowth of obstructive tissue longer than one year postoperative |
| Incontinence | N39.3 | stress incontinence |
|  | N39.4 | other specified urinary incontinence |
|  | N39.41 | urge incontinence |
|  | N39.42 | incontinence without sensory awareness |
| Hematuria | 599.7 | hematuria, unspecified |
|  | R31.9 |  |
|  | R31.0 | gross hematuria |
| Urinary retention | R33.9 |  |
|  | 788.20 | Retention of urine, unspecified |
|  | 788.21 | Incomplete bladder emptying |
|  | 788.29 | Other abnormality of the urinary bladder |
| BPH diagnosis | N40.0 | (BPH w/o LUTS) |
|  | N40.1 | (BPH w/ LUTS) |
|  | N40.2 | Nodular prostate w/o LUTS |
|  | N40.3 | Nodular prostate w/ LUTS |
|  | 600.0 | Hypertrophy (benign) of prostate |
|  | 600.9 | Hyperplasia (benign) of prostate w/o urinary obstruction |
|  | N32.0 | bladder-neck obstruction |
|  | N13.9 | obstructive and reflex uropathy, unspecified |
| Catheter supplies | A4311-6, A4353-4 | Indwelling catheters |
|  | A4310-6, A5102, A5112 | Catheter insertion trey |
|  | A4314-6, A4354, A4357-8, A5102, A5112 | Urinary drainage collection system |
|  | A4351-2 | Intermittent catheter |
